# Short-term effects of controlled mating and selection on the genetic variance of honeybee populations

**DOI:** 10.1038/s41437-021-00411-2

**Published:** 2021-03-30

**Authors:** Manuel Du, Richard Bernstein, Andreas Hoppe, Kaspar Bienefeld

**Affiliations:** Institute for Bee Research Hohen Neuendorf, Hohen Neuendorf, Germany

**Keywords:** Bioinformatics, Genetic models, Quantitative trait, Animal breeding, Genetic variation

## Abstract

Directional selection in a population yields reduced genetic variance due to the Bulmer effect. While this effect has been thoroughly investigated in mammals, it is poorly studied in social insects with biological peculiarities such as haplo-diploidy or the collective expression of traits. In addition to the natural adaptation to climate change, parasites, and pesticides, honeybees increasingly experience artificial selection pressure through modern breeding programs. Besides selection, many honeybee breeding schemes introduce controlled mating. We investigated which individual effects selection and controlled mating have on genetic variance. We derived formulas to describe short-term changes of genetic variance in honeybee populations and conducted computer simulations to confirm them. Thereby, we found that the changes in genetic variance depend on whether the variance is measured between queens (inheritance criterion), worker groups (selection criterion), or both (performance criterion). All three criteria showed reduced genetic variance under selection. In the selection and performance criteria, our formulas and simulations showed an increased genetic variance through controlled mating. This newly described effect counterbalanced and occasionally outweighed the Bulmer effect. It could not be observed in the inheritance criterion. A good understanding of the different notions of genetic variance in honeybees, therefore, appears crucial to interpreting population parameters correctly.

## Introduction

Many populations of social insects are currently exposed to intense levels of natural selection due to climate change, pesticides, habitat fragmentation, or parasites (Chapman and Bourke 2001; Le Conte and Navajas 2008; Mikheyev et al. 2015). These factors greatly influence the genetic structure of these populations. For many populations of the European honeybee (*Apis mellifera*), additional genetic changes are to be expected due to artificial selection, since great efforts have been undertaken to establish modern breeding programs for honeybees in Europe (Büchler et al. 2013; Uzunov et al. 2015). It is known that intensified selection leads to a rapid reduction of genetic variance due to a disequilibrium effect which was first described by Bulmer (1971). This Bulmer effect appears for both natural (Hawthorne 1998) and artificial selection (Atkins and Thompson 1986). For diploid dioecious species, it can be explained as follows.

Assume a purely additive heritable trait that follows the assumptions of Fisher’s infinitesimal model (Fisher 1918). Further, assume a population with discrete generations in which every individual *z* has a true-breeding value *u*_*z*_ with respect to that trait. Under panmixia and the absence of selection (assuming that effects of inbreeding, mutation, and genetic drift are negligible), the variance of breeding values within one generation *g* of the population is precisely the additive genetic variance ${\sigma }_{A}^{2}$ of the trait:

1 $${\mathrm{var}}({u}_{z}| z\,{\mathrm{is}}\,{\mathrm{in}}\,{\mathrm{generation}}\,g)={\sigma }_{A}^{2}.$$

The breeding value of an individual *z* is inherited from its sire *s*(*z*) and dam *d*(*z*) via the formula

2 $${u}_{z}=\frac{1}{2}\cdot {u}_{s(z)}+\frac{1}{2}\cdot {u}_{d(z)}+{\delta }_{s(z)}+{\delta }_{d(z)},$$

where the Mendelian sampling terms *δ*_*s*(*z*)_ and *δ*_*d*(*z*)_ are independent of *u*_*s*(*z*)_ and *u*_*d*(*z*)_ and one another, and each has variance $\frac{1}{4}\cdot {\sigma }_{A}^{2}$. Passing to variances in Eq. (2) thus yields

3 $$\begin{array}{l}{\rm{var}}\left({u}_{z}| z\ {\rm{is}} \, {\mathrm{in}} \, {\mathrm{generation}}\ g+1\right)\\ = \frac{1}{4}\cdot {\rm{var}}({u}_{s}| s \, {\rm{is}} \, {\mathrm{sire}} \, {\mathrm{in}} \, {\mathrm{generation}} \, g)\\ +\frac{1}{4}\cdot {\rm{var}}({u}_{d}| d \, {\rm{is}} \, {\mathrm{dam}} \, {\mathrm{in}} \, {\mathrm{generation}} \, g)\\ +\frac{1}{2}\cdot {\rm{cov}}({u}_{s},{u}_{d}| s,d\ {\rm{are}} \, {\mathrm{parents}} \, {\mathrm{in}} \, {\mathrm{generation}} \, g)\\ +\frac{1}{2}\cdot {\sigma }_{A}^{2}.\end{array}$$

In the absence of selection or assortative mating, the dams and sires in generation *g* are independent random samples of the population, whence their variances are again $\sigma^2_A$ and their covariance is 0. In this way, Eq. (3) illustrates how in ideal populations the additive genetic variance remains constant over generations. In the presence of selection, however, while the variances of the Mendelian sampling terms *δ*_*s*(*z*)_ and *δ*_*d*(*z*)_ remain unchanged, the sires and dams in generation *g* are no longer a representative sample of the population and their variance will differ. If the genetic variance within the selected sires and dams in generation *g* are $(1-{\kappa }_{s})\cdot {\sigma }_{A}^{2}$ and $(1-{\kappa }_{d})\cdot {\sigma }_{A}^{2}$, respectively, the variance in generation *g* + 1 will be

4 $${\rm{var}}({u}_{z}| z \, {\rm{is}} \, {\mathrm{in}} \, {\mathrm{generation}} \, g+1)=\left(1-\frac{{\kappa }_{s}+{\kappa }_{d}}{4}\right)\cdot {\sigma }_{A}^{2}.$$

As *κ*_*s*_ and *κ*_*d*_ are usually positive, Eq. (4) means a reduction of genetic variance within the population as compared to the case without selection. However, it should be noted that this reduction of variance (unlike reductions caused by drift or inbreeding) is reversible. Once the selection regime ends, the original variance will soon be restored (Bulmer 1971). Similar considerations can be made if a non-zero covariance between parental breeding values is imposed by assortative mating (Tallis and Leppard 1987). If selection is performed as truncation selection with estimated breeding values on a normally distributed trait, *κ*_*j*_ (=*κ*_*s*_ or *κ*_*d*_) can be quantified as

5 $${\kappa }_{j}={i}_{j}({i}_{j}-{x}_{j})\cdot {\rho }_{j}^{2},$$

where *i*_*j*_ denotes the sex-specific selection intensity, *x*_*j*_ the standardized truncation point, and *ρ*_*j*_ the correlation between estimated and true breeding values of sires and dams, respectively (Dekkers 1992). For shorter notation, the term *i*_*j*_(*i*_*j*_ − *x*_*j*_) is commonly abbreviated by *k*_*j*_ (van der Werf and de Boer 1990).

The biology of social insects, and in particular of honeybees, varies from other species in a number of ways which prevent the theory explained above from carrying over immediately. The most notable differences are the haplo-diploid genetics, the mating behavior, and the expression of selection traits in a colony rather than in individuals. Honeybees separate into three castes: queens, workers, and drones. While the male drones are haploid and develop from unfertilized eggs, queens and workers are female and diploid; however, in general, only drones and queens can reproduce. A colony consists of one queen and up to 40,000 workers, all of which are daughters of the queen. Depending on the season, the queen can further produce several hundreds of drones. All practical work in- and outside the hive is carried out by workers and queen, often in close collaboration, whence most economic traits in honeybees are assumed to have maternal (queen) and direct (worker group) effects (Bienefeld and Pirchner 1991; Brascamp et al. 2016; Chevalet and Cornuet 1982). While queens and drones are generally seen as individuals, workers are usually only regarded as a collective. Shortly after hatching, a newly-born queen undertakes her nuptial flight during which she mates in the air with drones from other colonies. The semen stored during this flight is used later on to fertilize eggs to develop into female offspring. The mating procedure is usually not observable, and artificial selection on the paternal path is therefore not straightforward and often abstained from.

One possibility to enable selection on the paternal path in breeding schemes is isolated mating stations. These consist of secluded geographic areas, like valleys or islands, which are void of honeybee colonies with the exception of a group of colonies that are held there for the purpose of producing drones. Usually, the queens of these drone producing colonies share a common dam, which in turn has been selected for her superior breeding values. The mating stations fulfill two purposes: (a) ensure that only drones with favorable genetic features are involved in the mating process, and (b) provide pedigree information on the paternal side and therefore lead to more accurate breeding value estimations. In honeybee pedigrees, mating stations correspond to sires in pedigrees of other farm animals. Thus, mating stations are also referred to as *pseudo sires* (Bienefeld et al. 2007, 1989; Brascamp and Bijma 2014; Plate et al. 2019a).

Simulation studies have shown that breeding schemes with controlled mating are superior to those without (Plate et al. 2019b). Ideally, the introduction of a new breeding scheme for a honeybee population, therefore, involves both selection and controlled mating for one or several traits and establishing controlled mating (Uzunov et al. 2017). However, there are cases, where selection is performed without controlled mating (Andonov et al. 2019; Pernal et al. 2012; Spivak and Reuter 2001). In the context of conservation programs, situations are also conceivable where mating is controlled but no directional selection is imposed.

Both controlled mating and selection may have short-term implications on the variance structure within the honeybee population which to our knowledge have not been analyzed so far. In this article, we give a theoretical derivation of the short-term effects to be expected upon installation of a new honeybee breeding program. Furthermore, we carried out simulation studies with the program BeeSim (Plate et al. 2019a) to verify and quantify our results.

## Theory

When it comes to the inheritance of breeding values in honeybees, three different pathways have to be distinguished, depending on whether the recipient is a queen, a drone, or a worker group (Plate et al. 2019a). Throughout, for a queen *q*, we denote by *Q*(*q*) her dam queen and by *D*(*q*) her sire drone. Likewise, let *Q*(*d*) be the dam queen of a drone *d*. Finally, for a worker group *w*, let *Q*(*w*) be the dam queen and let ${D}_{1}(w),...,{D}_{{n}_{D}}(w)$ be the sire drones, i.e., the drones which *Q*(*w*) mated with. (See Table 1 for an overview of the used variables.) Most breeding traits in honeybees possess maternal and direct effects, which generally have different additive genetic variances ${\sigma }_{A,m}^{2}$ and ${\sigma }_{A,d}^{2}$, respectively, as well as a covariance *σ*_*A*,*md*_, which usually takes on negative values (Bienefeld and Pirchner 1991; Brascamp et al. 2016). While direct breeding values are only expressed in worker groups and maternal breeding values are only expressed in queens, all individuals possess true breeding values for both effects. To acknowledge this, we denote breeding values of queens, drones, or worker groups (**u**_*q*_, **u**_*d*_, and ${\bar{{\bf{u}}}}_{w}$) as vectors $${{\bf{u}}}_{z}=\left[\begin{array}{l}{u}_{z}^{{\rm{mat}}}\\ {u}_{z}^{{\rm{dir}}}\end{array}\right]$$. Breeding values of worker groups (${\bar{{\bf{u}}}}_{w}$) are equipped with a bar to signify that they are average values of the individual workers of a colony. The additive genetic variance is denoted as a matrix $${{\bf{V}}}_{A}=\left[\begin{array}{ll}{\sigma }_{A,m}^{2}&{\sigma }_{A,md}\\ {\sigma }_{A,md}&{\sigma }_{A,d}^{2}\end{array}\right]$$. With this notation, we have for a queen *q*:

6 $${{\bf{u}}}_{q}=\frac{1}{2}\cdot {{\bf{u}}}_{Q(q)}+{{\bf{u}}}_{D(q)}+{{\boldsymbol{\delta }}}_{Q(q)},$$

where the Mendelian sampling term ***δ***_*Q*(*q*)_ has variance $\frac{1}{4}\cdot {{\bf{V}}}_{A}$. Note that since drones are haploid, they pass on all of their genetic information and there is no Mendelian sampling among their gametes. For a drone *d*, we have:

7 $${{\bf{u}}}_{d}=\frac{1}{2}\cdot {{\bf{u}}}_{Q(d)}+{{\boldsymbol{\delta }}}_{Q(d)},$$

where again the Mendelian sampling term ***δ***_*Q*(*d*)_ has variance $\frac{1}{4}\cdot {{\bf{V}}}_{A}$. Finally, for a worker group *w*:

8 $${\bar{{\bf{u}}}}_{w}=\frac{1}{2}\cdot {{\bf{u}}}_{Q(w)}+\frac{1}{{n}_{D}}\cdot \mathop{\sum }\limits_{i = 1}^{{n}_{D}}{{\bf{u}}}_{{D}_{i}(w)}.$$

There is no Mendelian sampling term in the inheritance to worker groups because the breeding values of worker groups are means over a large number of individual workers. Equation (8) makes the simplifying assumption that all drones contribute the same relative number of workers, which is not guaranteed in reality.

**Table 1:** Notation key.

*a*_DPQ_	Average relationship between drone producing queens on a mating station
${C}_{p,{a}_{{\rm{DPQ}}}}$	$=\frac{1}{4}\cdot (p+(1-p){a}_{{\rm{DPQ}}})$; genetic covariance of drones on a mating station
**c**_*w*_	$={\rm{cov}}({\hat{I}}_{w},{\bar{{\bf{u}}}}_{w})^{\prime}$; covariance of selection index and workers’ breeding values
*D*(*q*)	Sire drone of a queen *q*
${D}_{1}(w),...,{D}_{{n}_{D}}(w)$	Sire drones of a worker group *w*
***δ***_*Q*_	Mendelian sampling in the inheritance from a queen *Q*
${h}_{m}^{2}$, ${h}_{d}^{2}$	Maternal and direct heritability, resp.
${\hat{I}}_{w}$	$={\hat{\bar{u}}}_{w}^{{\rm{mat}}}+{\hat{\bar{u}}}_{w}^{{\rm{dir}}}$; selection index
${i}_{{\hat{I}}_{w}}$	Selection intensity of index selection
${k}_{{\hat{I}}_{w}}$	$={i}_{{\hat{I}}_{w}}({i}_{{\hat{I}}_{w}}-{x}_{{\hat{I}}_{w}})$; reduction factor of selection index variance under selection
*n*_*D*_	Number of drones a queen mates with
*n*_*d*_, *n*_*s*_	Number of dams of breeding queens and drone producing queens per year, resp.
*p*	Probability of two drones on a mating station to share the same dam queen
*Q*(*q*), *Q*(*w*), *Q*(*d*)	Dam queen of a queen *q*, worker group *w*, or drone *d*, resp.
*r*_*md*_	Genetic correlation between maternal and direct effects
${\sigma }_{A,m}^{2}$, ${\sigma }_{A,d}^{2}$	Maternal and direct additive genetic variance, resp.
*σ*_*A*,*md*_	Additive genetic covariance between maternal and direct effects
${\sigma }_{E}^{2}$	Residual variance
${\sigma }_{{\hat{I}}_{w}}^{2}$	Variance of selection index
*t*_cont_, *t*_sel_	Time of introduction of controlled mating and selection, resp.
**u**_*q*_, ${\bar{{\bf{u}}}}_{w}$, **u**_*d*_	True breeding value of a queen *q*, worker group *w*, or drone *d*, resp.
${\hat{{\bf{u}}}}_{q}$, ${\hat{\bar{{\bf{u}}}}}_{w}$	(BLUP)-estimated breeding value of a queen *q*, or worker group *w*, resp.
**V**_*A*_	Additive genetic variance matrix
${x}_{{\hat{I}}_{w}}$	Standardized truncation point of index selection

We start our investigation of the additive genetic variance within a honeybee population considering the queens of that population. Passing to variances in Eq. (6) yields

9 $$\begin{array}{ll}{\mathrm{var}}({{\bf{u}}}_{q}| q\ {\rm{is}}\;{\mathrm{queen}})=\,\frac{1}{4}\cdot {\mathrm{var}}({{\bf{u}}}_{Q(q)}| q\ {\rm{is}}\;{\mathrm{queen}})\\ \,+\,{\mathrm{var}}({{\bf{u}}}_{D(q)}| q\ {\rm{is}}\;{\mathrm{queen}})\\ \,+\,{\mathrm{cov}}({{\bf{u}}}_{Q(q)},{{\bf{u}}}_{D(q)}| q\ {\rm{is}}\;{\mathrm{queen}})\\ \,+\,\frac{1}{4}\cdot {{\bf{V}}}_{A}.\end{array}$$

The variance in breeding values for drones is derived from Eq. (7):

10 $${\mathrm{var}}({{\bf{u}}}_{d}| d\ {\rm{is}}\;{\mathrm{drone}})=\frac{1}{4}\cdot {\mathrm{var}}({{\bf{u}}}_{Q(d)}| d\ {\rm{is}}\;{\mathrm{drone}})+\frac{1}{4}\cdot {{\bf{V}}}_{A}.$$

Turning to the worker groups, we assume for simplicity that every queen mates with the same number *n*_*D*_ of drones. Consequently, passing to variances in Eq. (8) yields

11 $$\begin{array}{ll}{\rm{var}}\left({\bar{{\bf{u}}}}_{w}| w\ {\rm{is}}\;{\mathrm{worker}}\;{\mathrm{group}}\right)\\ = \frac{1}{4}\cdot {\mathrm{var}}({{\bf{u}}}_{Q(w)}| w\ {\rm{is}}\;{\mathrm{worker}}\;{\mathrm{group}})\\ + \, {\mathrm{cov}}({{\bf{u}}}_{Q(w)},{{\bf{u}}}_{{D}_{i}(w)}| w\ {\rm{is}}\;{\mathrm{worker}}\;{\mathrm{group}})\\ + \, \frac{1}{{n}_{D}}\cdot {\mathrm{var}}({{\bf{u}}}_{{D}_{i}(w)}| w\ {\rm{is}}\;{\mathrm{worker}}\;{\mathrm{group}})\\ + \, \frac{{n}_{D}-1}{{n}_{D}}\cdot {\mathrm{cov}}({{\bf{u}}}_{{D}_{i}(w)},{{\bf{u}}}_{{D}_{j}(w)}| w\ {\rm{is}}\;{\mathrm{worker}}\;{\mathrm{group}},\ i\,\ne \,j).\end{array}$$

Equations (9)–(11) may be assumed for any honeybee population. To draw further conclusions, it is necessary to make additional assumptions on the population structure. In particular, it plays a role if mating is controlled or uncontrolled and whether or not there is selection.

### Uncontrolled mating, no selection

We first consider the case that no selection is employed and mating always occurs uncontrolledly. In this case, dam queens and sire drones of queens are independent random samples of the entire queen and drone population, respectively, whence in Eq. (9), the terms “var(**u**_*Q*(*q*)_∣*q* is queen)” and “var(**u**_*D*(*q*)_∣*q* is queen)” may be replaced by “var(**u**_*q*_∣*q* is queen)” and “var(**u**_*d*_∣*d* is drone)”, respectively, and the covariance term vanishes. Likewise, all queens have the same chance to become the dam of a drone, whence the term “var(**u**_*Q*(*d*)_∣*d* is drone)” in Eq. (10) is identical with “var(**u**_*q*_∣*q* is queen)”.

Combining the thus modified Eqs. (9) and (10) yields

12 $$\begin{array}{ll}{\rm{var}}({{\bf{u}}}_{q}| q\ {\rm{is}}\;{\mathrm{queen}})=\frac{1}{4}\cdot {\rm{var}}({{\bf{u}}}_{q}| q\ {\rm{is}}\;{\mathrm{queen}})\\ +\frac{1}{4}\cdot {\rm{var}}({{\bf{u}}}_{q}| q\ {\rm{is}}\;{\mathrm{queen}})+\frac{1}{4}\cdot {{\bf{V}}}_{A}+\frac{1}{4}\cdot {{\bf{V}}}_{A}\\ =\frac{1}{2}\cdot {\rm{var}}({{\bf{u}}}_{q}| q\ {\rm{is}}\;{\mathrm{queen}})+\frac{1}{2}\cdot {{\bf{V}}}_{A}.\end{array}$$

This shows in analogy to Eq. (3), how the genetic variance in the queen population remains constantly **V**_*A*_:

13 $${\rm{var}}({{\bf{u}}}_{q}| q\ {\rm{is}}\;{\mathrm{queen}})={{\bf{V}}}_{A}.$$

As a consequence, we obtain from Eq. (10)

14 $${\rm{var}}({{\bf{u}}}_{d}| d\ {\rm{is}}\;{\mathrm{drone}})=\frac{1}{2}\cdot {{\bf{V}}}_{A}.$$

Like in the inheritance to queens, the covariance terms also vanish in the inheritance to worker groups (Eq. (11)). Since the sire drones of worker groups form a random sample of all drones, we obtain from Eqs. (13) and (14):

15 $${\rm{var}}({\bar{{\bf{u}}}}_{w}| w\ {\rm{is}}\;{\mathrm{worker}}\;{\mathrm{group}})=\left(\frac{1}{4}+\frac{1}{2{n}_{D}}\right)\cdot {{\bf{V}}}_{A}.$$

### Controlled mating without selection

The situation that mating on isolated mating stations is imposed but no directional selection is carried out is very rare in honeybee breeding in reality. Nevertheless, in very small populations, like that of the Sicilian honeybee (*A. m. siciliana*), which are endangered by introgression of other subspecies and where the effective population size is too small to allow for directional selection, it may be a viable conservation strategy (Muñoz et al. 2014).

In this case, Eqs. (13) and (14) remain unchanged. The reason is that, while sire drones and drone producing queens are no longer random samples of the population, they still are unselected descendants of the dams of the drone producing queens which in turn are randomly chosen among the population.

But when it comes to the variance structure of worker groups, differences appear in the term ${\rm{cov}}({{\bf{u}}}_{{D}_{i}(w)},{{\bf{u}}}_{{D}_{j}(w)})$, as the drones which are involved in a controlled mating are generally related, whence there are positive covariances between their breeding values. For two drones *D*_*i*_(*w*) ≠ *D*_*j*_(*w*) involved in a controlled mating, we may assume a probability *p* that they descend from the same drone producing queen and probability 1 − *p* that their dams are different queens on the same mating station. Usually, this probability *p* is assumed to depend only on the number of drones producing queens on a mating station and potentially the number *n*_*D*_ of drones that mate with a queen (Brascamp and Bijma 2014). In consequence, we have for *D*_*i*_(*w*) ≠ *D*_*j*_(*w*):

16 $$\begin{array}{ll}{\rm{cov}}({{\bf{u}}}_{{D}_{i}(w)},{{\bf{u}}}_{{D}_{j}(w)})\\={\rm{cov}}(\frac{1}{2}\cdot {{\bf{u}}}_{Q({D}_{i}(w))}+{{\boldsymbol{\delta }}}_{Q({D}_{i}(w))},\frac{1}{2}\cdot {{\bf{u}}}_{Q({D}_{j}(w))}+{{\boldsymbol{\delta }}}_{Q({D}_{j}(w))})\\ =\frac{1}{4}\cdot {\rm{cov}}({{\bf{u}}}_{Q({D}_{i}(w))},{{\bf{u}}}_{Q({D}_{j}(w))})\\ =\frac{1}{4}\cdot (p+(1-p){a}_{{\rm{DPQ}}})\cdot {{\bf{V}}}_{A},\end{array}$$

where *a*_DPQ_ denotes the average relationship between drone producing queens on a mating station. For shorter notation, we will denote this covariance term by

17 $${C}_{p,{a}_{{\rm{DPQ}}}}:=\frac{1}{4}\cdot (p+(1-p){a}_{{\rm{DPQ}}}).$$

From Eq. (16), we conclude in the case of controlled mating:

18 $${\rm{var}}({\bar{{\bf{u}}}}_{w}| {w}\ {\text{is worker group}})=\left(\frac{1}{4}+\frac{1}{2{n}_{D}}+\frac{{n}_{D}-1}{{n}_{D}}\cdot {C}_{p,{a}_{{\mathrm{DPQ}}}}\right)\cdot {{\bf{V}}}_{A}.$$

The values of *p* and *a*_DPQ_ depend on assumptions on the composition of drone producing queens on a mating station, the distribution of offspring to drones and queens, and the history of the breeding program (Brascamp and Bijma 2014, 2019). However, for reasonable assumptions (a sister group of eight drone producing queens on a mating station, each queen mates with 12 drones), one can estimate *p* ≈ 0.125 (Bienefeld et al. 2007) and *a*_DPQ_ ≈ 0.32 (Brascamp and Bijma 2014, 2019). Assuming these values, we arrive at the estimate that the introduction of controlled mating without selection can increase the variance of true breeding values of worker groups by around 31.8%.

### Influence of selection

As in other species, the introduction of selection has an influence on the genetic variance of the population. Truncation selection in honeybees is generally imposed as follows: by best linear unbiased prediction (BLUP) breeding value estimation (Henderson 1975), estimated breeding values ${\hat{{\bf{u}}}}_{q}$ and ${\hat{\bar{{\bf{u}}}}}_{w}$ for queens and worker groups are determined and fertilized queens are then selected based on the index ${\hat{I}}_{w}={\hat{\bar{u}}}_{w}^{{\rm{mat}}}+{\hat{\bar{u}}}_{w}^{{\rm{dir}}}$, formed as the sum of the estimated maternal and direct breeding values of their worker groups. The changes of the variance structure of the population due to selection can be described in terms of three key variables: the selection intensity ${i}_{{\hat{I}}_{w}}$, with which the selection index is selected, the variance ${\sigma }_{\hat{{I}_{w}}}^{2}$ of the selection index, and the vector of covariances $${{\bf{c}}}_{w}=\left[\begin{array}{l}{\rm{cov}}({\bar{u}}_{w}^{{\rm{mat}}},{\hat{I}}_{w})\\ {\rm{cov}}({\bar{u}}_{w}^{{\rm{dir}}},{\hat{I}}_{w})\end{array}\right]$$ between the true maternal and direct breeding values of the worker groups and the selection index.

To derive these dependencies, we build on a general fact about normally distributed random vectors which was first formulated by Pearson (1903) and reviewed in modern notation by e.g., Gianola et al. (1989): let **y** and **w** be jointly normally distributed characteristics of a population with variance matrix $${\rm{var}}\left(\left[\begin{array}{l}{\bf{y}}\\ {\bf{w}}\end{array}\right]\right)=\left[\begin{array}{ll}{{\bf{V}}}_{{\bf{yy}}}&{{\bf{V}}}_{{\bf{yw}}}\\ {{\bf{V}}}_{{\bf{wy}}}&{{\bf{V}}}_{{\bf{ww}}}\end{array}\right]$$. If then, by the selection of some individuals, the variance structure regarding **y** is changed to ${{\bf{V}}}_{{\bf{yy}}}^{{\rm{sel}}}$, the variance structure regarding **w** can be expected to change to

19 $${{\bf{V}}}_{{\bf{ww}}}^{{\rm{sel}}}={{\bf{V}}}_{{\bf{ww}}}-{{\bf{V}}}_{{\bf{wy}}}{{\bf{V}}}_{{\bf{yy}}}^{-1}({{\bf{V}}}_{{\bf{yy}}}-{{\bf{V}}}_{{\bf{yy}}}^{{\rm{sel}}}){{\bf{V}}}_{{\bf{yy}}}^{-1}{{\bf{V}}}_{{\bf{yw}}}.$$

For a queen *Q* with worker group *w*, the BLUP-estimated breeding value ${\hat{\bar{{\bf{u}}}}}_{w}$ of the worker group coincides with the estimated breeding value ${\hat{{\bf{u}}}}_{q}$ of an unphenotyped daughter queen *q* of *Q* (Brascamp and Bijma 2019). Likewise, the vector **c**_*w*_ coincides with the vector of covariances of the maternal and direct true breeding values of *q* with the selection index ${\hat{I}}_{w}$ (proof given in Appendix A). Therefore, under the absence of selection, we have for a worker group *w* and queen *q* with *Q*(*w*) = *Q*(*q*) the joint variance structure

20 $${\rm{var}}\left(\left[\begin{array}{l}{\hat{I}}_{w}\\ {{\bf{u}}}_{q}\end{array}\right]\right)=\left[\begin{array}{ll}{\sigma }_{{\hat{I}}_{w}}^{2}&{{\bf{c}}}_{w}^{\prime}\\ {{\bf{c}}}_{w}&{{\bf{V}}}_{A}\end{array}\right].$$

Truncation selection with intensity ${i}_{{\hat{I}}_{w}}$ will reduce ${\rm{var}}({\hat{I}}_{w})$ to

21 $${\sigma }_{{\hat{I}}_{w},{\rm{sel}}}^{2}={i}_{{\hat{I}}_{w}}({i}_{{\hat{I}}_{w}}-{x}_{{\hat{I}}_{w}})\cdot {\sigma }_{{\hat{I}}_{w}}^{2},$$

where, as in Eq. (5), ${x}_{{\hat{I}}_{w}}$ denotes the standardized truncation point. Consequently, by Eq. (19) and using the abbreviation ${k}_{{\hat{I}}_{w}}:={i}_{{\hat{I}}_{w}}({i}_{{\hat{I}}_{w}}-{x}_{{\hat{I}}_{w}})$, the variance of **u**_*q*_ among the next generation of queens is

22 $${\rm{var}}{({{\bf{u}}}_{q})}_{{\rm{sel}}}={{\bf{V}}}_{A}-\frac{{k}_{{\hat{I}}_{w}}}{{\sigma }_{{\hat{I}}_{w}}^{2}}\cdot {{\bf{c}}}_{w}{{\bf{c}}}_{w}^{\prime}.$$

Equation (22) corresponds to the reduction of genetic variance under index selection in other livestock species as it is written explicitly by e.g., Bijma and Rutten (2002).

In standard theory on the Bulmer effect, the reduction of genetic variance in the population is inferred from the variance among the selected parents (cf. Eq. (4)). For honeybees, we circumvented this by the use of Pearson’s formula (19). It is, however, also possible to calculate the genetic variances and covariances among the selected queens and the drones they mated with. We present these calculations in Appendix B. Of the results derived there, is noteworthy that even in the absence of assortative mating there is a nonzero covariance between the true breeding values of selected queens and the drones they mated with. This covariance is negative definite, reflecting that a mediocre queen will only be selected if it is mated with excellent drone material, whereas a queen of exceptional quality may still be selected if she is mated with drones of lower quality.

The consequences of selection for the genetic variances among drones and worker groups are omitted in this manuscript but can be worked out following similar lines as those specified in Appendix B.

### Notions of total genetic variance

In order to assess and judge the status of a honeybee population regarding genetic variance in practice, it is necessary to condense the different variances of direct and maternal effects for the members of the three castes into one notion of total additive genetic variance. In practical applications, focus mostly is not laid on individual queens or worker groups; instead, colonies consisting of a queen and her offspring workers are seen as the relevant entities. For these, different combinations of direct and maternal breeding values are considered to be of interest. The sum of direct and maternal breeding values of a queen, ${u}_{q}^{{\rm{mat}}}+{u}_{q}^{{\rm{dir}}}$, is the so-called inheritance criterion (IC). Its variance structure shows the possibilities of genetic inheritance to future generations (Brascamp and Bijma 2019). The performance criterion (PC) is the sum of the maternal breeding value of a queen and the direct breeding value of her worker group, ${u}_{Q(w)}^{{\rm{mat}}}+{\bar{u}}_{w}^{{\rm{dir}}}$, and describes the genetic contribution to the phenotype of a colony (Plate et al. 2019a). Finally, the sum of direct and maternal breeding values of a worker group, ${\bar{u}}_{w}^{{\rm{mat}}}+{\bar{u}}_{w}^{{\rm{dir}}}$, is the so-called selection criterion (SC), because the estimation of this value determines whether a queen will be selected for reproduction (Brascamp and Bijma 2014; Plate et al. 2019a).

The theory developed above implies that the introduction of controlled mating increases the variance in the performance and selection criteria (because these consider worker groups), but leaves the IC unaffected. The introduction of selection decreases the genetic variance of a honeybee population in all three criteria (IC, PC, and SC). In the following, we confirm and quantify these theoretical findings with the help of a computer simulation study.

## Methods

**Fig. 1:**
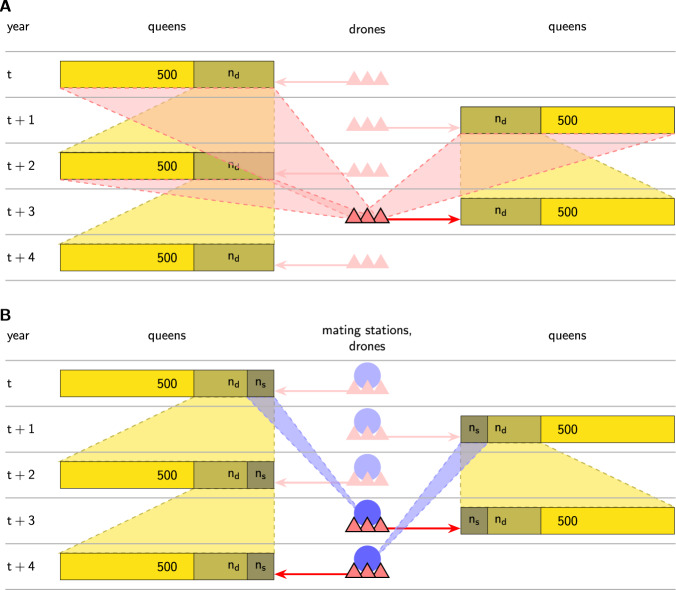
Scheme of reproduction. **A** Scheme of reproduction under uncontrolled mating. Dams of queens are 2 years old and selected either randomly or by BLUP breeding value estimation. Dams of drones are 1–3 years old and are randomly selected. **B** Scheme of reproduction under controlled mating. Dams of queens are 2 years old and grand dams of drones are 3 years old. Both are selected either randomly or by BLUP breeding value estimation. This figure is inspired by Figure 1 in Plate et al. (2019a).

**Fig. 2:**
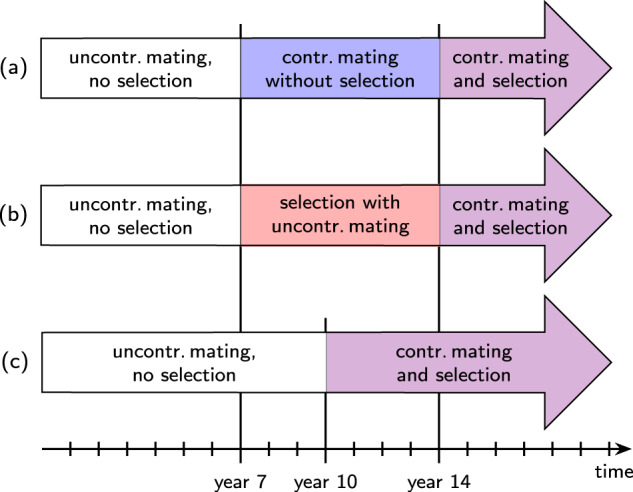
Breeding schemes. The three different simulated set-ups regarding the order of introduction of controlled mating and selection: (**a**) controlled mating introduced before selection, (**b**) selection introduced before controlled mating, and (**c**) simultaneous introduction of controlled mating and selection.

We used the program BeeSim (Plate et al. 2019a) to simulate a honeybee population consisting of 500 colonies per year over the course of 20 years. All queens, drones, and worker groups were simulated individually and inherited a trait following the infinitesimal model according to Eqs. (6)–(8). For the first years, no selection was carried out, and queens mated uncontrolledly. Uncontrolled mating was realized as in Plate et al. (2019b); i.e., the drones were provided by randomly chosen queens of ages between 1 and 3 years (see Fig. 1A). Starting from year *t*_cont_, mating took place on isolated mating stations. From year *t*_sel_ on, the reproducing queens were selected after a BLUP breeding value estimation like previously simulated in (Plate et al. 2019a, 2020). We simulated three different situations regarding the introduction of controlled mating and selection (see Fig. 2): (a) first controlled mating, then BLUP selection: (*t*_cont_ = 7, *t*_sel_ = 14), (b) first selection, then controlled mating (*t*_cont_ = 14, *t*_sel_ = 7), and (c) simultaneous start of controlled mating and BLUP selection (*t*_cont_ = *t*_sel_ = 10).

Throughout the simulations, in each year *n*_*d*_ of the 2-year-old queens of that year were chosen to serve as dams for the next generation, producing 500/*n*_*d*_ offspring queens each (see Fig. 1). While that choice was random up to year *t*_sel_ − 1, from year *t*_sel_ on, the queens were chosen by truncation selection based on BLUP breeding values which were obtained by BLUPF90 (Misztal et al. 2002) with the use of a honeybee specific relationship matrix (Bernstein et al. 2018; Brascamp and Bijma 2014). We simulated the different values of *n*_*d*_ = 50, *n*_*d*_ = 100, and *n*_*d*_ = 250. In the years with BLUP selection, different values of *n*_*d*_ represent different selection intensities on the maternal path.

In the years with controlled mating (from *t*_cont_ on), *n*_*s*_ of the 3-year-old queens were chosen to serve as dams of the drone producing queens of one mating station each (see Fig. 1B). In case of no selection, these queens were chosen at random, but from year *t*_sel_ on, they were chosen based on BLUP truncation selection. We simulated the different values *n*_*s*_ = 5, *n*_*s*_ = 25, and *n*_*s*_ = 50 to represent different selection intensities on the paternal path. Each mating station comprised eight drone producing queens. Regardless if mating was controlled or uncontrolled, all queens mated with *n*_*D*_ = 12 drones.

Finally, we made two different choices for the selection trait, which differed mainly in the strength of the negative correlation between direct and maternal effects. For both traits, we chose a maternal additive genetic variance of ${\sigma }_{A,m}^{2}=1$, a direct additive genetic variance of ${\sigma }_{A,d}^{2}=2$, and a residual variance of ${\sigma }_{E}^{2}=1$. Then in one case we chose a moderate negative covariance of *σ*_*A*,*md*_ = −0.5, and in the other case a stronger covariance of *σ*_*A*,*md*_ = −1. The former trait yields a maternal heritability of ${h}_{m}^{2}=0.46$, a direct heritability of ${h}_{d}^{2}=0.31$, and a correlation between effects of *r*_*md*_ = −0.35. The corresponding values for the latter trait are ${h}_{m}^{2}=0.60$, ${h}_{d}^{2}=0.40$, and *r*_*md*_ = −0.71 (see Brascamp and Bijma (2019) for a detailed description of the calculation of heritabilities for honeybees).

The different combinations of choices for *t*_cont_, *t*_sel_, *n*_*d*_, *n*_*s*_, and *σ*_*A*,*md*_ made up a total of 54 different simulation set-ups. The simulations for each set-up were repeated 200 times for stable results. To assess the changes in genetic variance, we compared the values attained in the year before the introduction of a new breeding strategy with the results from the year of introduction; i.e., genetic variance levels of years *t*_cont_ − 1 and *t*_sel_ − 1 were compared with those of years *t*_cont_ and *t*_sel_, respectively.

## Results

**Fig. 3:**
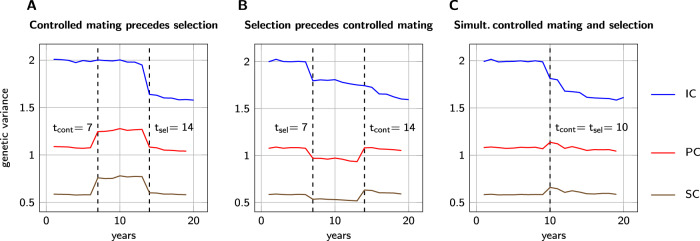
Genetic variance. Changes of different notions of genetic variance in a honeybee population following the introduction of controlled mating and/or BLUP selection. Results are shown for the parameters *n*_*d*_ = 100, *n*_*s*_ = 25, and *r*_*md*_ = −0.35. **A** Controlled mating introduced before selection. **B** Selection introduced before controlled mating. **C** Simultaneous introduction of controlled mating and selection.

The simulations corroborated the theoretical findings. The introduction of controlled mating increased the population variance in the PC and the SC which are (partly) determined by the worker groups but had little effect on the IC which is measured only in the queens. Meanwhile, the introduction of selection caused a reduction of variance in all criteria (see Fig. 3). A similar, though less pronounced, the pattern could be observed for the direct and maternal effects individually (see Supplementary Fig. S1). In the following, we present our findings in greater detail.

### Introduction of controlled mating

Independent of the order of introduction of controlled mating and selection, controlled mating did practically not affect the genetic variance in the IC. Upon introduction of controlled mating, reductions or increases in this criterion were mostly below 1% and did not show any clear direction (see Table 2 and Fig. 3A, B). The increase of genetic variance in the PC due to the introduction of controlled mating depended heavily on the selection trait. For the trait with moderate correlation between effects, *r*_*md*_ = −0.35, there was an increase between 13.4 and 15.2%; for the trait with strong correlation, *r*_*md*_ = −0.71, the effect was significantly stronger: between 26.4 and 31.2%. Finally, the SC showed similar behavior in both traits. If controlled mating was introduced before selection, it caused an increase of genetic variance of between 24.7 and 27.4%; if it was introduced after selection the increase of variance was only between 20.3 and 24.4%. In the breeding schemes which introduced controlled mating after selection, the increase of genetic variance in the SC was slightly higher, when the negative correlation between maternal and direct effects was strong, *r*_*md*_ = −0.71. There was no clear indication of an effect of the number of dam queens on the intensity of the variance increase (see Table 2).

**Table 2:** Change (in %) of population variance after the introduction of controlled mating.

		Controlled mating before selection			Selection before controlled mating		
		50 dams	100 dams	250 dams	50 dams	100 dams	250 dams
*r*_*md*_ = −0.35	IC	−1.0	+0.1	+0.5	−0.6	−0.6	−0.2
	PC	+13.4	+14.1	+15.0	+14.9	+15.2	+14.1
	SC	+24.7	+26.2	+27.0	+20.5	+20.9	+20.3
*r*_*md*_ = −0.71	IC	+0.5	−0.4	+0.1	+0.4	−0.7	−0.1
	PC	+27.0	+27.2	+27.0	+26.4	+30.2	+28.6
	SC	+27.4	+26.4	+27.1	+21.8	+24.4	+23.8

Relative change (in %) of genetic variance in the three different criteria IC, PC, and SC, after the introduction of controlled mating. Results are shown for different correlations *r*_*md*_ between direct and maternal effects and different numbers of selected dams per year.

**Fig. 4:**
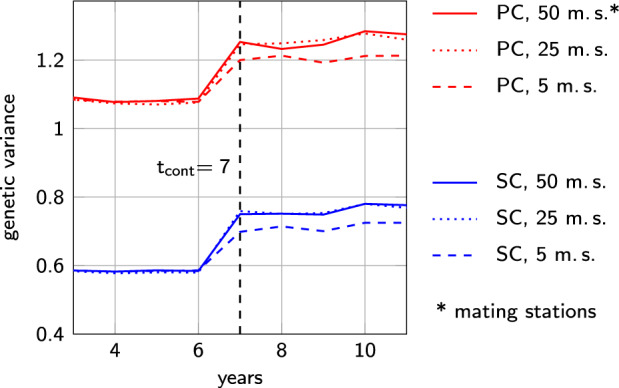
Influence of the number of pseudo sires on the increase of genetic variance in the PC and SC. Development of genetic variance with the introduction of controlled mating without selection in year *t*_sel_ = 7. Results are shown for *n*_*d*_ = 100 and *r*_*md*_ = −0.35.

The introduction of controlled mating yielded significantly different results for breeding schemes with only five pseudo sires from those with 25 or 50 pseudo sires. With only five pseudo sires the increase of genetic variance in PC and SC was significantly lower than in breeding schemes with more pseudo sires. The increase of variance in both criteria was reduced by roughly a fifth with slight variations between the different traits and selection schemes (see Fig. 4).

### Introduction of selection

When the selection was introduced after imposing controlled mating (*t*_cont_ = 7, *t*_sel_ = 14), it caused a decrease of variance in the population in all variance criteria (IC, PC, and SC) of between 8.8 and 21.2% (see Table 3 and Fig. 3A). When the introduction of selection preceded the introduction of controlled mating (*t*_cont_ = 14, *t*_sel_ = 7), the reduction of variance appeared less severe, between 3.2 and 10.4% (see also Fig. 3B). The reduction was more pronounced for the trait with a moderate negative correlation between effects, *r*_*md*_ = −0.35 than for the trait with the stronger negative correlation, *r*_*md*_ = −0.71. Furthermore, a higher selection intensity (fewer dams selected) resulted in a stronger decrease of variance within the population. When the selection was introduced after controlled mating, the Bulmer effect showed most strongly in the SC, followed by IC and PC. In the case of *t*_cont_ > *t*_sel_, the opposite order could be observed: the Bulmer effect affected the PC strongest and the SC weakest. The number of mating stations, i.e., pseudo sires, *n*_*s*_, had only a minor effect on the results and there was no clear tendency indicating whether a higher number of pseudo sires caused a stronger or weaker Bulmer effect (data not shown). Furthermore, in the case of selection preceding controlled mating, we observed a significant reduction of genetic variance in the IC from year 15 to year 16 by between 3.2 and 7.6% which was mainly caused by a reduced variance of direct breeding values of queens (see Fig. 3B and Supplementary Fig. S1).

**Table 3:** Change (in %) of population variance through the introduction of selection.

		Controlled mating before selection			Selection before controlled mating		
		50 dams	100 dams	250 dams	50 dams	100 dams	250 dams
*r*_*md*_ = −0.35	IC	−18.4	−16.4	−14.3	−9.9	−9.5	−7.6
	PC	−16.6	−14.6	−13.6	−10.4	−10.2	−8.1
	SC	−22.6	−21.2	−20.7	−8.3	−8.0	−6.6
*r*_*md*_ = −0.71	IC	−13.9	−11.7	−9.0	−5.2	−4.7	−3.7
	PC	−13.6	−10.2	−8.8	−6.8	−6.0	−5.2
	SC	−18.7	−16.3	−14.4	−4.3	−4.0	−3.2

Reduction (in %) of genetic variance in the three different criteria IC, PC, and SC, due to the Bulmer effect upon introduction of BLUP selection. Results are shown for different correlations *r*_*md*_ between direct and maternal effects and different numbers of selected dams per year.

### Combined effects

The simultaneous introduction of controlled mating and selection (*t*_sel_ = *t*_cont_ = 10) resulted in an immediate reduction of genetic variance in the IC of 6.9–11.3% (*r*_*md*_ = −0.35), and of 3.1–6.2% (*r*_*md*_ = −0.71), respectively. Two years after the introduction of the breeding scheme, a second drop in genetic variance occurred with a reduction of 6.6–8.6% (*r*_*md*_ = −0.35), and of 3.9–7.2% (*r*_*md*_ = −0.71), respectively (see Fig. 3C).

In the performance and selection criteria, we observed an initial increase of variance by up to 14.1% (*r*_*md*_ = −0.35) and 26.2% (*r*_*md*_ = −0.71), respectively. Subsequently, however, the level of genetic variance in these criteria decreased and thus undermined the initial gain (see Fig. 3C). Comparing the genetic variance of year 9 (directly before the introduction of the breeding scheme) with those of year 13 we could still see an increase of genetic variance for *r*_*md*_ = −0.71, while for *r*_*md*_ = −0.35 there were some selection schemes with a positive effect and some with a negative effect on the genetic variance in the PC and SC (see Table 4).

**Table 4:** Change (in %) of population variance by the simultaneous introduction of controlled mating and selection.

		*r*_*md*_ = −0.35			*r*_*md*_ = −0.71		
		IC	PC	SC	IC	PC	SC
5 pseudo sires	50 dams	−17.5	−6.1	−2.0	−12.8	+11.1	+7.4
	100 dams	−16.3	−2.1	+2.2	−12.0	+12.4	+6.4
	250 dams	−14.7	−1.8	+1.2	−10.1	+12.2	+8.3
25 pseudo sires	50 dams	−18.0	−2.6	+3.9	−11.8	+16.6	+12.5
	100 dams	−15.9	+2.1	+7.0	−10.4	+22.4	+15.2
	250 dams	−14.1	+1.5	+7.3	−8.0	+21.4	+17.5
50 pseudo sires	50 dams	−18.1	−1.7	+4.2	−11.8	+19.8	+14.9
	100 dams	−16.4	+1.1	+6.6	−10.0	+23.3	+16.5
	250 dams	−13.4	+3.3	+9.5	−7.9	+26.9	+19.3

Relative change (in %) of genetic variance from year 9 to year 13, when both controlled mating and selection were introduced in year 10.

## Discussion

### Significance of the notions of genetic variance

The different criteria for assessing the genetic variance within a honeybee population (IC, PC, and SC) showed different behavior upon the introduction of breeding schemes, which entails the question of which criterion is the most relevant for honeybee breeding.

Traditionally, the Bulmer effect has mainly been seen as a limitation to the genetic progress a population can reach in total (Dempfle 1974). In this regard, the IC is the most relevant, because only queens can reproduce and thus contribute to the genetic progress of future generations. The IC corresponds to the notion of *heritable variance* in the presence of indirect genetic effects in other agricultural species (Bijma 2011; Brascamp and Bijma 2019). As we have shown, the population variance in this criterion was reduced by the introduction of selection and largely unaffected by the introduction of controlled mating. Therefore, we assume that when new breeding schemes are introduced in honeybees, the Bulmer effect has mainly the same consequences for the breeding success as in other agricultural species.

The other two notions of genetic variance (PC and SC) have more indirect effects on breeding success. The PC influences the phenotypic variance of a population. An increase of genetic variance in the PC without an increase of variance in the IC does not increase the evolutionary potential of a population. However, in a study on microbia, van Boxtel et al. (2017) showed that non-inheritable phenotypic variance can be powerful insurance against sudden extinction of (sub-)populations. Breeding decisions in honeybees are based on the SC. Increased variance in this criterion, like it is caused by the introduction of controlled mating, allows BLUP to yield estimated breeding values with higher accuracy. Consequently, the higher genetic variance in the SC is a minor contributor to the superiority of breeding schemes with controlled mating over those without (Plate et al. 2019b).

### Changes of genetic variance

Overall, the simulation results confirmed the theoretical predictions, i.e., that the introduction of selection decreases the genetic variance within the population in all criteria (IC, PC, and SC), while the introduction of controlled mating results in an increase of genetic variance in those criteria which account for worker groups (PC and SC) but does not influence the variance structure of the queens in the population (IC). In the context of honeybees, possible consequences of the Bulmer effect have been mentioned by Moritz (1986), but only under the a priori assumption that it behaves like in other agricultural species. To our knowledge, this is the first thorough investigation of the Bulmer effect for honeybees. In addition, the positive effect of controlled mating on the variance in the PC and SC is described for the first time.

#### Introduction of controlled mating

Both theory and simulation studies agreed that the introduction of controlled mating increases the genetic variance in the PC and SC but not in the IC. In the situation where controlled mating is imposed on unselected populations, theory and simulations for the SC also confirm each other quantitatively, with the realized increase of genetic variance being only slightly lower than the 31.8% we derived in the theory section.

In selected populations, the increase of genetic variance in the PC and SC in the course of the introduction of controlled mating was lower than in unselected populations (see Table 2 and Fig. 3A, B), because the variance among the dams of the drone producing queens was reduced. Plate et al. (2019b) showed that controlled mating improves the accuracy of breeding value estimation for honeybees. The enhanced accuracy of breeding values after the introduction of controlled mating leads to higher covariances **c**_*w*_ between the selection index and the true breeding values and thus to a further reduction of genetic variance among the dams of future generations according to Eq. (22). This explains the continued reduction of genetic variance over several years after the introduction of controlled mating which was especially pronounced when selection and controlled mating were introduced simultaneously (see Fig. 3C).

The effect of increased variance between worker groups due to controlled mating is to some extent comparable to the effect of assortative mating in other agricultural species as described by Tallis and Leppard (1987, 1988). If parents are mated assortatively, the positive correlation between their breeding values results in an increased genetic variance. In the case of controlled mating of honeybees, the increased genetic variance between worker groups is likewise caused by a positive correlation of the parental breeding values (albeit not between the breeding values of sire and dam but between the multiple sire drones of a worker group).

Another possible interpretation of the described effect is that of a shift of genetic variance. While the genetic variance between worker groups is increased, controlled mating decreases the intracolonial genetic variance (Oldroyd et al. 1992). In fact, much as it is the case for queens, the genetic variance between all individual worker bees in the population remains unaffected by controlled mating.

#### Introduction of selection

The reduction of genetic variance in the population after the start of selection was significantly stronger when mating was controlled (see Table 3 and Fig. 3A, B). This has two reasons: Controlled mating allows (a) for selection on the paternal path which causes a reduction of variance among the sire drones, and (b) for a more accurate breeding value estimation and thus a further reduced variance among dam queens (Plate et al. 2019b). The different rates of variance reduction for the two traits can be explained by the different accuracies of BLUP selection. Stronger negative covariances between effects have been shown to lead to lower accuracy in the breeding value estimation of honeybees and other agricultural species (Plate et al. 2019a, b; Roehe and Kennedy 1993). In consequence, there is more genetic variance among the selected dams (and in the case of controlled mating also sires) and thus a less pronounced Bulmer effect. Similarly, truncation selection schemes with fewer selected dams show a greater similarity among the selected individuals and thus a stronger Bulmer effect. The number of pseudo sires has a twofold effect. On the one hand, a small number of pseudo sires means a high intensity of selection (large ${k}_{\hat{{I}_{w}}}$), but on the other hand, Plate et al. (2019b) have shown that small numbers of mating stations also reduce the accuracy of the BLUP breeding value estimation (small values in **c**_*w*_). Equation (22) indicates that these two aspects have opposing effects on the genetic variance. The effect of the number of pseudo sires on the Bulmer effect due to the introduction of selection is therefore involved and depends on cofactors such as genetic parameters and the number of selected dams.

### Limitations of theory

#### Inhomogeneous populations

In our simulations, we assumed a very homogeneous population in which the population size and the number of selected dams remained constant each year, and each dam mated with the same number *n*_*D*_ of drones. In particular, the last assumption is unrealistic unless all queens are artificially inseminated. This is slightly problematic since the putatively constant number *n*_*D*_ is explicitly used in the derivation of the formulas for the variance structure of worker groups (Eq. (11) and derived equations). A natural approach for populations in which queens mate with different numbers of drones would be to use the average number of drones mated to a queen, ${\overline{n}}_{D}$, for which estimates have been published (Tarpy and Nielsen 2002). While this is probably acceptable in most situations, it should be mentioned that the replacement of *n*_*D*_ by ${\overline{n}}_{D}$ in formulas derived from Eq. (11) will systematically underestimate the genetic variance among worker groups in heterogeneous populations. This is a consequence of Jensen’s inequality (Jensen 1906) since the inversion of *n*_*D*_ in Eq. (11) is a convex function.

#### Small numbers of dams and sires

We assumed that the genetic variance within an unselected sample of the population equals the population variance. In fact, the expected sample variance in a sample of size *n* is lower than the population variance by a factor $\frac{n-1}{n}$ (Kenney and Keeping 1957). The derived formulas should therefore be used with care if there are only a few pseudo sires or if very few dam queens are selected. In our simulations, an effect of reduced sample variance due to the small sample size could be observed in simulations with only five pseudo sires (Fig. 4). The lower increase of genetic variance in the PC and SC in these cases can be explained by the reduced variance within the sample of the five dams of the drone producing queens on mating stations.

#### Middle to long term effects

Besides the Bulmer effect, changes in genetic variance can also be caused by genetic drift, inbreeding, and mutation. The effects of these factors on honeybees have been investigated in various studies (Beye et al. 2006; Plate et al. 2019a, 2020; Zayed and Packer 2005). However, they will influence the genetic variance only over an extended period of time and not in the short time frame considered in the present study. The initial gain of genetic variance in the performance and SC after the introduction of controlled mating could therefore become reduced over time as the selection of pseudo sires increases the risks of accumulated inbreeding and genetic drift (Plate et al. 2019a, b, 2020). Furthermore, we mainly quantified the effects of controlled mating and selection to the immediate next generation. In other livestock species, it has been shown that the Bulmer effect can further decrease the genetic variance over several generations until an equilibrium is reached (Van Grevenhof et al. 2012). But following a geometric series, convergence to equilibrium is generally fast and Fig. 3A, B suggests that no large effect occurs in later generations since the curves turn flat immediately after the disruptions in years *t*_cont_ and *t*_sel_.

#### Genetic model

Like the original work of Bulmer (1971), this study assumes throughout that selection traits follow the infinitesimal model, which is unlikely in reality. Plate et al. (2019a) showed that the choice of genetic model is of little relevance for the short-term genetic gain in simulated honeybee breeding systems. However, this may be different in the context of genetic variance. Turelli and Barton (1990) derived that short-term changes in genetic variance, such as the Bulmer effect, may behave differently if the selection trait deviates from normality or in the presence of linkage disequilibrium in the unselected population. The high recombination rate for the honeybee genome (Beye et al. 2006) may alleviate such effects.

### Other selection schemes

#### Within-family selection

In our simulations, we used a truncation selection scheme based on BLUP breeding values. For other agricultural species, it has been shown that the reduction of genetic variance due to the Bulmer effect is greatly reduced or entirely absent if within-family selection is applied (Dempfle 1974; Wei et al. 1996). These results can be expected to hold true for honeybees as far as the reduction of genetic variance due to selection is concerned.

In reality, honeybee breeding programs usually realize neither pure truncation selection nor pure within-family selection but a complicated blend of selection systems shaped by heterogeneous decisions of breeders. Furthermore, some breeding systems are open for the introduction of queens with unregistered dams which also affects the variance structure (Brascamp et al. 2016).

#### Artificial insemination

Artificial insemination of queens is a popular alternative to isolated mating stations (Cobey et al. 2013). In many cases, artificial insemination mimics the situation on a mating station, i.e., drones for the insemination process are selected among colonies whose queens share a common dam. However, differing procedures are possible and sometimes applied in reality. Instead of using drones from several colonies, insemination of a queen can be performed with drones from a single colony (Gerula et al. 2014) or even with a single drone (Harbo 1999). If each queen is inseminated with drones from a single colony, the covariance terms in Eq. (11) will be increased and the increase of genetic variance in the PC and SC due to the controlled mating will be stronger. It will further intensify if the number of drones, *n*_*D*_, is reduced because this variable is divided by Eq. (11). In a hypothetical population in which all queens are inseminated by randomly selected single drones, Eq. (11) would simplify to

23 $${\rm{var}}({\bar{{\bf{u}}}}_{w}| w\ {\rm{is}}\;{\mathrm{worker}}\;{\mathrm{group}})=\frac{3}{4}\cdot {{\bf{V}}}_{A}.$$

As another extreme, queens can also be inseminated with semen from very large pools of unrelated drones (Pieplow et al. 2017). If no selection is imposed on the drone sires, Eq. (15) can be used to describe the variance structure among the colonies. However, since the number of drones is very high, *n*_*D*_ can be assumed to tend to infinity, reducing Eq. (15) to

24 $${\rm{var}}({\bar{{\bf{u}}}}_{w}| w\ {\rm{is}}\;{\mathrm{worker}}\;{\mathrm{group}})=\frac{1}{4}\cdot {{\bf{V}}}_{A}.$$

Equations (23) and (24) describe the maximum and minimum genetic variance among worker groups which can be attained through different insemination schemes (not taking genetic drift, inbreeding, or mutations into account).

### Supplementary information

Supplementary Figure S1

## Data Availability

The dataset used and analyzed in this article is available at 10.5061/dryad.pzgmsbck4. The source code of the simulation program BeeSim is available at 10.5061/dryad.1nh544n.

## Appendix A

### Covariance of selection index and breeding values of replacement queens

Let *w* be a worker group and *q* be an unphenotyped queen with *Q*(*w*) = *Q*(*q*). Let ${\bar{{\bf{u}}}}_{w}$, **u**_*q*_ and ${\hat{\bar{{\bf{u}}}}}_{w}$, ${\hat{{\bf{u}}}}_{q}$ be their respective true and BLUP-estimated breeding values. In this appendix, we will show that

25 $${\rm{cov}}({\bar{{\bf{u}}}}_{w},{\hat{\bar{u}}}_{w}^{{\rm{mat}}}+{\hat{\bar{u}}}_{w}^{{\rm{dir}}})={\rm{cov}}({{\bf{u}}}_{q},{\hat{\bar{u}}}_{w}^{{\rm{mat}}}+{\hat{\bar{u}}}_{w}^{{\rm{dir}}}).$$

#### Proof

It is a well-established fact that the estimated breeding values for *w* and *q* coincide (Brascamp and Bijma 2019, Equation (8)):

26 $${\hat{\bar{{\bf{u}}}}}_{w}={\hat{{\bf{u}}}}_{q}.$$

Furthermore, it is a property of every BLUP-estimate $\hat{{\bf{u}}}$ of a true-breeding value **u** that (Henderson 1975)

27 $${\rm{cov}}({\bf{u}},\hat{{\bf{u}}})={\rm{var}}(\hat{{\bf{u}}}).$$

Therefore:

$$\begin{array}{l}{\rm{cov}}({\bar{{\bf{u}}}}_{w},{\hat{\bar{u}}}_{w}^{{\rm{mat}}}+{\hat{\bar{u}}}_{w}^{{\rm{dir}}})={\rm{cov}}({\bar{{\bf{u}}}}_{w},{\hat{\bar{{\bf{u}}}}}_{w})\left[\begin{array}{l}1\\ 1\end{array}\right]\mathop{=}\limits^{(27)}{\rm{var}}({\hat{\bar{{\bf{u}}}}}_{w})\left[\begin{array}{l}1\\ 1\end{array}\right]\mathop{=}\limits^{(26)}{\rm{var}}({\hat{{\bf{u}}}}_{q})\left[\begin{array}{l}1\\ 1\end{array}\right]\\ \mathop{=}\limits^{(27)}{\rm{cov}}({{\bf{u}}}_{q},{\hat{{\bf{u}}}}_{q})\left[\begin{array}{l}1\\ 1\end{array}\right]\mathop{=}\limits^{(26)}{\rm{cov}}({{\bf{u}}}_{q},{\hat{\bar{{\bf{u}}}}}_{w})\left[\begin{array}{l}1\\ 1\end{array}\right]={\rm{cov}}({{\bf{u}}}_{q},{\hat{\bar{u}}}_{w}^{{\rm{mat}}}+{\hat{\bar{u}}}_{w}^{{\rm{dir}}}).\end{array}$$

q.e.d.

## Appendix B

### Genetic (co-)variances among selected queens and drones

In this appendix, we expound how BLUP-selection in honeybees affects the genetic (co-)variances among the selected queens and the drones they mated with. The notation is the same as in the main article.

In the absence of selection, the selection index ${\hat{I}}_{w}$ and the true breeding values of worker groups ${\bar{{\bf{u}}}}_{w}$ have the joint variance

28 $${\rm{var}}\left(\left[\begin{array}{l}{\hat{I}}_{w}\\ {\bar{{\bf{u}}}}_{w}\end{array}\right]\right)=\left[\begin{array}{ll}{\sigma }_{{\hat{I}}_{w}}^{2}&{{\bf{c}}}_{w}^{\prime}\\ {{\bf{c}}}_{w}&W\cdot {{\bf{V}}}_{A}\end{array}\right],$$

where $W={W}_{u}:=\frac{1}{4}+\frac{1}{2{n}_{D}}$ if mating is uncontrolled (Eq. (15)) and $W={W}_{c}:=\frac{1}{4}+\frac{1}{2{n}_{D}}+\frac{{n}_{D}-1}{{n}_{D}}\cdot {C}_{p,{a}_{{\rm{DPQ}}}}$ if mating is controlled (Eq. (18)). Truncation selection will reduce ${\rm{var}}({\hat{I}}_{w})$ to ${k}_{{\hat{I}}_{w}}\cdot {\sigma }_{{\hat{I}}_{w}}^{2}$, whence by Pearson’s Eq. (19), the genetic variance among the worker groups of selected queens is

29 $${\rm{var}}{({\bar{{\bf{u}}}}_{w})}_{{\rm{sel}}}=W\cdot {{\bf{V}}}_{A}-\frac{{k}_{{\hat{I}}_{w}}}{{\sigma }_{{\hat{I}}_{w}}^{2}}\cdot {{\bf{c}}}_{w}{{\bf{c}}}_{w}^{\prime}.$$

To conclude the changes of (co-)variances among the true breeding values of selected queens and the drones they mated with, we apply Eq. (19) to the joint variance matrix of the vector ${\bf{u}}:=[{\bar{{\bf{u}}}}_{w}^{\prime}\ {{\bf{u}}}_{Q(w)}^{\prime}\ {{\bf{u}}}_{{D}_{1}(w)}^{\prime}\ \cdots \ {{\bf{u}}}_{{D}_{{n}_{D}}(w)}^{\prime}]^{\prime} :$

30 $${\rm{var}}({\bf{u}})=\left[\begin{array}{lllll}{\rm{var}}({\bar{{\bf{u}}}}_{w})&{\rm{cov}}({\bar{{\bf{u}}}}_{w},{{\bf{u}}}_{Q(w)})&{\rm{cov}}({\bar{{\bf{u}}}}_{w},{{\bf{u}}}_{{D}_{1}(w)})&\cdots \ &{\rm{cov}}({\bar{{\bf{u}}}}_{w},{{\bf{u}}}_{{D}_{{n}_{D}}(w)})\\ {\rm{cov}}({\bar{{\bf{u}}}}_{w},{{\bf{u}}}_{Q(w)})&{\rm{var}}({{\bf{u}}}_{Q(w)})&{\rm{cov}}({{\bf{u}}}_{Q(w)},{{\bf{u}}}_{{D}_{1}(w)})&\cdots \ &{\rm{cov}}({{\bf{u}}}_{Q(w)},{{\bf{u}}}_{{D}_{{n}_{D}}(w)})\\ {\rm{cov}}({\bar{{\bf{u}}}}_{w},{{\bf{u}}}_{{D}_{1}(w)})&{\rm{cov}}({{\bf{u}}}_{Q(w)},{{\bf{u}}}_{{D}_{1}(w)})&{\rm{var}}({{\bf{u}}}_{{D}_{1}(w)})&\cdots \ &{\rm{cov}}({{\bf{u}}}_{{D}_{1}(w)},{{\bf{u}}}_{{D}_{{n}_{D}}(w)})\\ \vdots &\vdots &\vdots &\ddots &\vdots \\ {\rm{cov}}({\bar{{\bf{u}}}}_{w},{{\bf{u}}}_{{D}_{{n}_{D}}(w)})&{\rm{cov}}({{\bf{u}}}_{Q(w)},{{\bf{u}}}_{{D}_{{n}_{D}}(w)})&{\rm{cov}}({{\bf{u}}}_{{D}_{1}(w)},{{\bf{u}}}_{{D}_{{n}_{D}}(w)})&\cdots \ &{\rm{var}}({{\bf{u}}}_{{D}_{{n}_{D}}(w)})\end{array}\right].$$

Under uncontrolled mating conditions, we have in the absence of selection

31 $$\begin{array}{ll}{\rm{cov}}({\bar{{\bf{u}}}}_{w},{{\bf{u}}}_{Q(w)})=\frac{1}{2}\cdot {{\bf{V}}}_{A},\\ {\rm{cov}}({\bar{{\bf{u}}}}_{w},{{\bf{u}}}_{{D}_{i}(w)})=\frac{1}{2{n}_{D}}\cdot {{\bf{V}}}_{A},\\ {\rm{var}}({{\bf{u}}}_{Q(w)})={{\bf{V}}}_{A},\\ {\rm{cov}}({{\bf{u}}}_{Q(w)},{{\bf{u}}}_{{D}_{i}(w)})={\bf{0}},\\ {\rm{var}}({{\bf{u}}}_{{D}_{i}(w)})=\frac{1}{2}\cdot {{\bf{V}}}_{A},\\ {\rm{cov}}({{\bf{u}}}_{{D}_{i}(w)},{{\bf{u}}}_{{D}_{j}(w)})={\bf{0}}.\end{array}$$

Inserting these values into Eq. (30) and applying the fact that the variance of true breeding values among the worker groups of selected queens is ${\rm{var}}{({\bar{{\bf{u}}}}_{w})}_{{\rm{sel}}}={W}_{u}\cdot {{\bf{V}}}_{A}-\frac{{k}_{{\hat{I}}_{w}}}{{\sigma }_{{\hat{I}}_{w}}^{2}}\cdot {{\bf{c}}}_{w}{{\bf{c}}}_{w}^{\prime}$ with ${W}_{u}=\frac{1}{4}+\frac{1}{2{n}_{D}}$ (Eq. (29)), we deduce by using Pearson’s Eq. (19):

32 $$\begin{array}{ll}{\rm{var}}{({{\bf{u}}}_{Q(w)})}_{{\rm{sel}}}={{\bf{V}}}_{A}-\frac{{k}_{{\hat{I}}_{w}}}{4{W}_{u}^{2}\cdot {\sigma }_{{\hat{I}}_{w}}^{2}}\cdot {{\bf{c}}}_{w}{{\bf{c}}}_{w}^{\prime},\\ {\rm{cov}}{({{\bf{u}}}_{Q(w)},{{\bf{u}}}_{{D}_{i}(w)})}_{{\rm{sel}}}=-\frac{{k}_{{\hat{I}}_{w}}}{4{W}_{u}^{2}\cdot {n}_{D}\cdot {\sigma }_{{\hat{I}}_{w}}^{2}}\cdot {{\bf{c}}}_{w}{{\bf{c}}}_{w}^{\prime},\\ {\rm{var}}{({{\bf{u}}}_{{D}_{i}(w)})}_{{\rm{sel}}}=\frac{1}{2}\cdot {{\bf{V}}}_{A}-\frac{{k}_{{\hat{I}}_{w}}}{4{W}_{u}^{2}\cdot {n}_{D}^{2}\cdot {\sigma }_{{\hat{I}}_{w}}^{2}}\cdot {{\bf{c}}}_{w}{{\bf{c}}}_{w}^{\prime},\\ {\rm{cov}}{({{\bf{u}}}_{{D}_{i}(w)},{{\bf{u}}}_{{D}_{j}(w)})}_{{\rm{sel}}}=-\frac{{k}_{{\hat{I}}_{w}}}{4{W}_{u}^{2}\cdot {n}_{D}^{2}\cdot {\sigma }_{{\hat{I}}_{w}}^{2}}\cdot {{\bf{c}}}_{w}{{\bf{c}}}_{w}^{\prime}.\end{array}$$

Under controlled mating conditions, we have ${W}_{c}=\frac{1}{4}+\frac{1}{2{n}_{D}}+\frac{{n}_{D}-1}{{n}_{D}}\cdot {C}_{p,{a}_{{\rm{DPQ}}}}$, and the analog to the set of Eq. (31) is

33 $$\begin{array}{ll}{\rm{cov}}({\bar{{\bf{u}}}}_{w},{{\bf{u}}}_{Q(w)})=\frac{1}{2}\cdot {{\bf{V}}}_{A},\\ {\rm{cov}}({\bar{{\bf{u}}}}_{w},{{\bf{u}}}_{{D}_{i}(w)})=\left({W}_{c}-\frac{1}{4}\right)\cdot {{\bf{V}}}_{A},\\ {\rm{var}}({{\bf{u}}}_{Q(w)})={{\bf{V}}}_{A},\\ {\rm{cov}}({{\bf{u}}}_{Q(w)},{{\bf{u}}}_{{D}_{i}(w)})={\bf{0}},\\ {\rm{var}}({{\bf{u}}}_{{D}_{i}(w)})=\frac{1}{2}\cdot {{\bf{V}}}_{A},\\ {\rm{cov}}({{\bf{u}}}_{{D}_{i}(w)},{{\bf{u}}}_{{D}_{j}(w)})={C}_{p,{a}_{{\rm{DPQ}}}}\cdot {{\bf{V}}}_{A},\end{array}$$

and with ${\rm{var}}{({\bar{{\bf{u}}}}_{w})}_{{\rm{sel}}}={W}_{c}\cdot {{\bf{V}}}_{A}-\frac{{k}_{{\hat{I}}_{w}}}{{\sigma }_{{\hat{I}}_{w}}^{2}}\cdot {{\bf{c}}}_{w}{{\bf{c}}}_{w}^{\prime}$, this yields

34 $$\begin{array}{ll}{\rm{var}}{({{\bf{u}}}_{Q(w)})}_{{\rm{sel}}}={{\bf{V}}}_{A}-\frac{{k}_{{\hat{I}}_{w}}}{4{W}_{c}^{2}\cdot {\sigma }_{{\hat{I}}_{w}}^{2}}\cdot {{\bf{c}}}_{w}{{\bf{c}}}_{w}^{\prime},\\ {\rm{cov}}{({{\bf{u}}}_{Q(w)},{{\bf{u}}}_{{D}_{i}(w)})}_{{\rm{sel}}}=-\frac{(4{W}_{c}-1)\cdot {k}_{{\hat{I}}_{w}}}{8{W}_{c}^{2}\cdot {\sigma }_{{\hat{I}}_{w}}^{2}}\cdot {{\bf{c}}}_{w}{{\bf{c}}}_{w}^{\prime},\\ {\rm{var}}{({{\bf{u}}}_{{D}_{i}(w)})}_{{\rm{sel}}}=\frac{1}{2}\cdot {{\bf{V}}}_{A}-\frac{{(4{W}_{c}-1)}^{2}\cdot {k}_{{\hat{I}}_{w}}}{16{W}_{c}^{2}\cdot {\sigma }_{{\hat{I}}_{w}}^{2}}\cdot {{\bf{c}}}_{w}{{\bf{c}}}_{w}^{\prime},\\ {\rm{cov}}{({{\bf{u}}}_{{D}_{i}(w)},{{\bf{u}}}_{{D}_{j}(w)})}_{{\rm{sel}}}={C}_{p,{a}_{{\rm{DPQ}}}}\cdot {{\bf{V}}}_{A}-\frac{{(4{W}_{c}-1)}^{2}\cdot {k}_{{\hat{I}}_{w}}}{16{W}_{c}^{2}\cdot {\sigma }_{{\hat{I}}_{w}}^{2}}\cdot {{\bf{c}}}_{w}{{\bf{c}}}_{w}^{\prime}.\end{array}$$

As an alternative to the derivation in the main article, the genetic variance among the next generation of queens (Eq. (22)) can also be obtained by inserting Eqs. (32) or (34) into Eq. (9).
