# Supplementary material for: Short-term effects of controlled mating and selection on the genetic variance of honeybee populations
Source: Heredity (Edinb). 2021 Mar 30;126(5):733–47. doi: 10.1038/s41437-021-00411-2 (PMC8102520; doi:10.1038/s41437-021-00411-2)
Supplement: Supplementary file 1 — Supplementary Figure S1 [file 41437_2021_411_MOESM1_ESM.pdf]

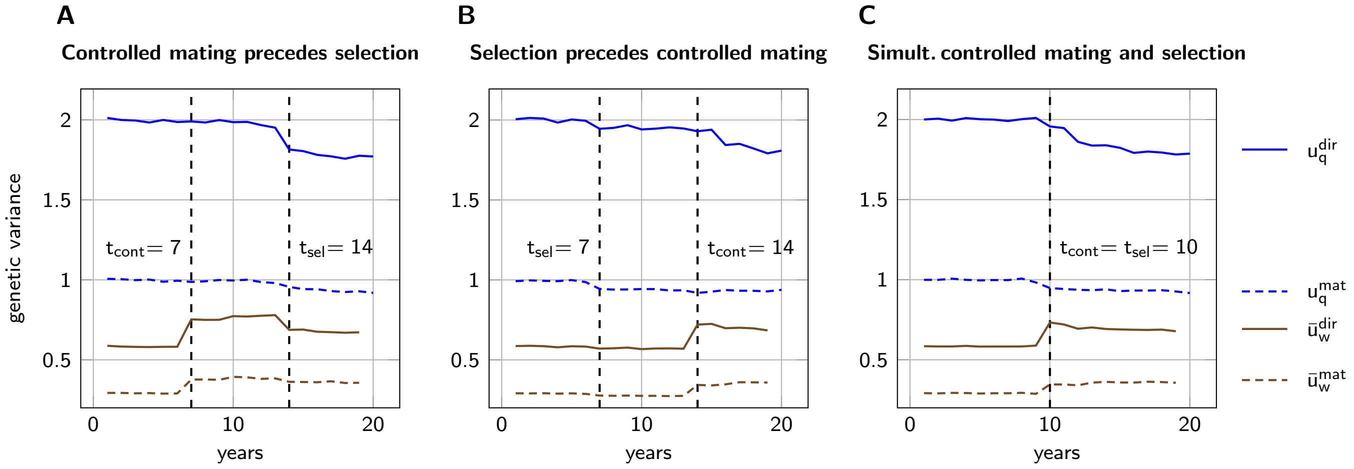

Supplementary Figure S1: **Genetic variance of maternal and direct effects.** Changes of maternal and direct genetic variances of queens and worker groups in a honeybee population following the introduction of controlled mating and/or BLUP selection. Results are shown for the parameters $n_d = 100$, $n_s = 25$, and $r_{md} = -0.35$. A: Controlled mating introduced before selection. B: Selection introduced before controlled mating. C: Simultaneous introduction of controlled mating and selection.
